# Paediatric HIV care in sub-Saharan Africa: clinical presentation and 2-year outcomes stratified by age group

**DOI:** 10.1111/tmi.12142

**Published:** 2013-06-20

**Authors:** Jihane Ben-Farhat, Marianne Gale, Elisabeth Szumilin, Suna Balkan, Elisabeth Poulet, Mar Pujades-Rodríguez

**Affiliations:** 1 Epicentre, Clinical Research DepartmentParis, France; 2 Médecins Sans FrontièresMelbourne, Australia; 3 Médecins Sans FrontièresParis, France; 4 University College LondonLondon, UK

**Keywords:** antiretroviral treatment, children, HIV, outcome assessment, Africa

## Abstract

**Objectives:**

To examine age differences in mortality and programme attrition amongst paediatric patients treated in four African HIV programmes.

**Methods:**

Longitudinal analysis of data from patients enrolled in HIV care. Two-year mortality and programme attrition rates per 1000 person-years stratified by age group (<2, 2–4 and 5–15 years) were calculated. Associations between outcomes and age and other individual-level factors were studied using multiple Cox proportional hazards (mortality) and Poisson (attrition) regression models.

**Results:**

Six thousand two hundred and sixty-one patients contributed 9500 person-years; 27.1% were aged <2 years, 30.1% were 2–4, and 42.8% were 5–14 years old. At programme entry, 45.3% were underweight and 12.6% were in clinical stage 4. The highest mortality and attrition rates (98.85 and 244.00 per 1000 person-years), and relative ratios (adjusted hazard ratio [aHR] = 1.92, 95% CI 1.56–2.37; incidence ratio [aIR] = 2.10, 95% CI 1.86–2.37, respectively, compared with the 5- to 14-year group) were observed amongst the youngest children. Increased mortality and attrition were also associated with advanced clinical stage, underweight and diagnosis of tuberculosis at programme entry.

**Conclusions:**

These results highlight the need to increase access, diagnose and provide early HIV care and to accelerate antiretroviral treatment initiation for those eligible. Adapted education and support for children and their families would also be important.

## Introduction

The provision of paediatric HIV care in resource-limited settings has expanded significantly over the last decade. At the end of 2010, 456 000 children were receiving combined antiretroviral therapy (ART) in low- and middle-income countries. However, treatment coverage amongst children requiring therapy remains low. In 2010, UNAIDS estimated that, globally, only 23% of children in need of ART received it, compared with 47% of adults (WHO 2011). Factors limiting the access of HIV-infected children to ART are multiple and include poor integration of HIV care with maternal and child health services, insufficient access to early infant diagnosis, implementation of strategies inefficient for retaining HIV-infected mothers and their children in care, and staff and caregivers who have often lacked of confidence and training to deal with children infected with HIV, especially during the early years of care provision when no adapted paediatric galenic antiretroviral formulations were available. Whilst progress in addressing these factors has been made over the last 10 years with initiatives, such as the development and commercialisation of paediatric fixed-dose combinations of antiretroviral drugs, the writing up of specific and increasingly simplified guidelines for the management of paediatric HIV infection training of health workers and, more recently, roll out of improved diagnostic technologies such as HIV deoxyribonucleic acid polymerase chain reaction (DNA PCR) using dried blood spot, substantial gaps in implementation exist.

Reassuringly, an increasing number of studies have reported treatment outcomes in children who are diagnosed and initiated on ART that are comparable, if not better than those of children treated in North America or Europe (Sutcliffe *et al.* 2008). Médecins Sans Frontières (MSF) began providing HIV care to adults in resource-limited settings in 2000 and to children in 2001. In this study, we examine the first 10 years of experience in treating paediatric HIV infection in sub-Saharan Africa by describing the clinical characteristics of children who enrolled in HIV care during this period and reporting treatment and programme outcomes 2 years after programme inclusion. Differences in patient mortality by age group and associations with other individual-level factors are also investigated to optimise patient management.

## Methods

### Study sites and population

The four HIV programmes supported by the French section of MSF were included in the evaluation: one from Uganda, one from Malawi and two from Kenya. Three were located in HIV clinics of district hospitals in rural areas, one of them with care highly decentralised to peripheral health centres, and were implemented in collaboration with the respective Ministries of Health, and one in an HIV clinic in a slum of Nairobi that was run by MSF alone. HIV care provision to children in these sites started between April 2001 and September 2002. All services related to HIV care, including laboratory testing, provision of combined antiretroviral therapy (ART), management of opportunistic infections and hospitalisation, were free.

For children aged >18 months, the diagnosis of HIV was based on the existence of two positive HIV rapid antibody tests. Younger children were diagnosed using HIV DNA PCR on dried blood spot when it was available and, when it was not, diagnosis was based on clinical and immunological criteria as recommended by the World Health Organization (WHO) and later confirmed with rapid antibody testing. Daily cotrimoxazole prophylaxis was prescribed, and ART eligibility was determined in accordance with WHO treatment guidelines. Medical visits for children ineligible for ART were generally scheduled every 3–6 months, or more frequently if clinically indicated. Visits for children receiving ART were scheduled at least monthly during the first 6 months and then every 2–3 months after clinical stabilisation. Group and individual counselling for caregivers and children, including therapeutic education and adherence support, were provided by peer workers and trained counsellors. Routine CD4 count testing was performed every 6–12 months before and after starting ART. Because of limited availability and cost constraints, viral load testing was reserved to cases of suspected treatment failure. However, in the clinic in Nairobi, routine viral monitoring was gradually introduced for children in 2007.

### Study design and data collection

All children (<15 years) who entered in one of the four HIV programmes between 8 April 2001 and 22 December 2010 and had attended two or more clinic visits were included in the analysis.

In agreement with the respective Ministries of Health, socio-demographic, clinico-immunological and treatment information were routinely recorded on medical files and entered into an electronic database (FUCHIA; Epicentre, Paris, France). Regular data consistency checks and verifications of key information (e.g. dates of birth, sex, date of ART start, CD4 cell count and percentage measures) were regularly performed on and off site to ensure the quality of the data.

### Statistical analysis

Patient characteristics at programme entry were described with summary statistics (proportions and medians with interquartile ranges), stratified by age group (<2, 2–4 and 5–15 years). Patient follow-up started at programme inclusion and was right-censored at the earliest of the following dates, transfer outside the HIV programme, last medical visit, or 24 months after programme entry. The duration of follow-up was split into three time periods: pre-ART, 0–6 and 6–24 months after ART start. Age-specific rates of mortality and programme attrition (deaths and lost to follow-up) expressed per 1000 person-years with 95% CI were calculated 3, 6, 12 and 24 months after programme entry. Patients lost to follow-up were those with a date of appointment not attended for more than 2 months, or patients with no date of next appointment not seen for more than 12 months (or for more than 6 months if they had started ART).

After verifying the proportional hazard assumption using the test of proportionality, multivariable Cox models were used to examine differences in 2-year mortality by age group. Crude and adjusted hazard ratios (HR) with 95% confidence interval (CI) were estimated. The following were factors considered in adjusted analyses: study site, sex, age group and year of programme entry (2001–2004, 2005–2007 and 2008–2010); baseline WHO clinical stage (1, 2, 3, 4 and missing), underweight status (no, yes and missing) and tuberculosis diagnosis; and period of follow-up (pre-ART, <6 months and 6–24 months after ART start). Underweight was defined as a weight-for-age score of <−2 for children aged <5 years old, and an age- and sex-specific body mass index (BMI) value equivalent to the <18.5 cut-off points of adults for children aged 5–14 years old (Cole *et al.* 2007). All individual-level factors associated with the outcome (*P* < 0.2) in univariable analysis were included in the final multivariable models. The likelihood ratio test for association was used, and the level of statistical significance considered was *P* < 0.05. The fit of the final models was assessed with the goodness-of-fit test for Cox.

Several sensitivity analyses were performed: first using competing risk analysis to account for the possible lack of independence between patients' risk of mortality and loss to follow-up; and second restricting analyses to patients with complete case data. The association between 2-year programme attrition and age was also investigated using multivariable Poisson regression using first information from all patients and then complete case data only. All analyses were carried out with Stata 11 (Stata Corp, College Station, Texas, USA).

## Results

### Patient characteristics

**Fig 1:**
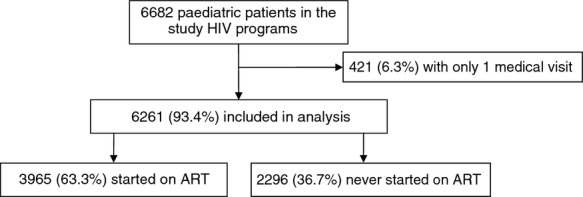
Flowchart of patients included in the analysis.

The analyses included the 6261 paediatric patients who had more than one recorded medical visit (Figure1) and contributed 9500 person-years of follow-up. Patient median age at programme entry was 4 years, with 27.1% of patients being aged <2 years, 30.1% aged 2–4 years and 42.8% aged 5–14 years (Table1). 51% of patients were females. The primary modes of entry in the programme were through voluntary counselling and testing (47.0%) or medical referral (42.7%). Less than 0.2% of children aged 2–14 years had history of prevention of mother-to-child transmission (PMTCT) prophylaxis use, whereas 5.9% of those in the <2-year age group did. And the number of young children with recorded PMTCT exposure gradually increased from 2005 to 2008 (5 and 32 children, respectively) and then slightly decreased again (down to 16 in 2010). Tuberculosis had been diagnosed in 9.8% of patients at the time of programme entry. Overall, 46.3% of patients presented in clinical stage 3 or 4, and 45.3% were underweight. No differences in severity of clinical presentation were observed across the three age groups, but tuberculosis was diagnosed more often after the first 2 years of age.

**Table 1:** Patient characteristics at programme entry stratified by age group

Characteristics	<2 years *N *=* *1698	2–4 years *N *=* *1885	5–14 years *N *=* *2678	Total *N *=* *6261
Female, *n* (%)	836 (49.2)	921 (48.9)	1440 (53.8)	3197 (51.1)
Median age, [IQR]	1.0 [0.4–1.4]	3.0 [2.3–4]	8.0 [6.3–11.0]	4.0 [1.8–7.7]
Mode of programme entry, *n* (%)				
Medical referral	629 (43.9)	715 (43.3)	965 (41.6)	2309 (42.7)
Volunteer counseling and testing	564 (39.4)	802 (48.5)	1175 (50.6)	2541 (47.0)
Other	240 (16.7)	136 (8.2)	182 (7.8)	558 (10.3)
Missing	265	232	356	853
History of PMTCT prophylaxis use, *n* (%)	100 (5.9)	3 (0.2)	3 (0.1)	106 (1.7)
Clinical stage, *n* (%)				
1	426 (41.7)	527 (35.2)	635 (29.4)	1588 (33.9)
2	150 (14.7)	273 (18.2)	505 (23.3)	928 (19.8)
3	323 (31.6)	512 (34.2)	741 (34.3)	1576 (33.7)
4	123 (12.0)	185 (12.4)	282 (13.0)	590 (12.6)
Missing	676	388	515	1579
Tuberculosis diagnosis, *n* (%)	126 (7.4)	197 (10.5)	290 (10.8)	613 (9.8)
Median weight, kg [IQR]	7.0 [5.7–8.6]	11.0 [9.0–13.0]	20.0 [16.0–24.5]	12.0 [8.5–19.0]
Missing, *n* (%)	3 (0.2)	3 (0.2)	5 (0.2)	11 (0.2)
Underweight, *n* (%)				
No	1015 (59.9)	954 (50.7)	1360 (54.1)	3329 (54.7)
Yes	680 (40.1)	928 (49.3)	1153 (45.9)	2761 (45.3)
Missing	3	3	165	171
Median CD4 cell count, cells/μl [IQR]	957 [544–1411]	706 [412–1063]	400 [189–682]	574 [299–974]
Missing, *n* (%)	864 (50.9)	615 (32.6)	814 (30.4)	2293 (36.6)
Median CD4 percentage [IQR]	17.0 [10.8–25.0]	17.4 [10.7–25.0]	13.0 [6.4–23.0]	16.2 [10.0–24.7]
Missing, *n* (%)	908 (53.5)	732 (38.8)	1990 (74.3)	3630 (58.0)

IQR, interquartile range; PMTCT, prevention of mother-to-child transmission of HIV infection.

### Mortality and programme attrition by age group

During the study period, 557 patients died and 1142 were lost to follow-up. Two-year mortality was higher in children aged <2 years than in older children (98.85 compared with 43.47 per 1000 person-years in the 2- to 4-year group; and 46.16 per 1000 person-years in the 5- to 14-year group). Adjusted HR (aHR) were 1.92, 95% CI 1.56–2.37 for the <2-year group and 0.87, 95% CI 0.69–1.10 for the 2- to 14-year group, compared with children aged 5–14 years (Table2). Similar results were obtained when competing risks models were used (adjusted sub-hazard ratio (aSHR) = 1.99, 95% CI 1.61–2.47 and aSHR = 0.96, 95% CI 0.76–1.21, respectively, compared with the 5- to 14-year group).

**Table 2:** Association between mortality and selected individual-level factors

Factors	No. of deaths	Mortality rate per 1000 PY	All patient data		Competing risks	Complete case
			Crude HR	Adjusted HR	Adjusted SHR	Adjusted HR
Site			*P *=* *0.13	*P *<* *0.001	*P *=* *0.02	*P *<* *0.001
Uganda	87	47.55 (38.54–58.67)	1	1	1	1
Malawi	325	60.94 (54.66–67.94)	1.30 (1.03–1.65)	1.18 (0.92–1.51)	1.28 (0.98–1.66)	1.69 (1.24–2.31)
Kenya – rural	82	61.58 (49.60–76.47)	1.32 (0.97–1.78)	0.89 (0.65–1.21)	0.89 (0.65–1.23)	1.07 (0.74–1.56)
Kenya – urban	63	62.63 (48.92–80.17)	1.32 (0.95–1.82)	1.23 (0.88–1.72)	1.30 (0.93–1.83)	1.58 (1.09–2.30)
Sex			*P *=* *0.01	*P *<* *0.001	*P *=* *0.02	*P *=* *0.06
Male	301	65.35 (58.37–73.16)	1	1	1	1
Female	256	52.31 (4628–59.13)	0.81 (0.68–0.95)	0.84 (0.71–1.00)	0.82 (0.69–0.97)	0.82 (0.67–1.01)
Age group			*P *<* *0.001	*P *<* *0.001	*P *<* *0.001	*P *<* *0.001
5–14 years	193	46.16 (40.01–53.16)	1	1	1	1
2–4 years	127	43.47 (36.53–51.73)	0.94 (0.75–1.18)	0.87 (0.69–1.10)	0.96 (0.76–1.21)	0.93 (0.72–1.21)
<2 years	237	98.85 (87.03–112.27)	2.07 (1.71–2.51)	1.92 (1.56–2.37)	1.99 (1.61–2.47)	1.83 (1.43–2.35)
Clinical stage			*P *<* *0.001	*P *<* *0.001	*P *=* *0.02	*P *=* *0.05
1	98	39.48 (32.39–48.12)	1	1	1	1
2	51	35.25 (26.79–46.38)	0.90 (0.64–1.26)	1.04 (0.74–1.46)	0.98 (0.70–1.37)	0.92 (0.66–1.30)
3	169	72.33 (62.21–84.10)	1.83 (1.43–2.35)	1.68 (1.28–2.20)	1.39 (1.06–1.83)	1.27 (0.96–1.68)
4	90	119.64 (97.31–147.10)	2.98 (2.24–3.97)	2.47 (1.80–3.38)	1.67 (1.20–2.33)	1.52 (1.08–2.13)
Missing	149	60.03 (51.13–70.49)	1.53 (1.19–1.98)	1.24 (0.95–1.61)	1.25 (0.96–1.64)	–
Tuberculosis diagnosis			*P *<* *0.001	*P *<* *0.001	*P *<* *0.001	*P *<* *0.001
No	453	52.39 (47.78–57.44)	1	1	1	1
Yes	104	121.89 (100.58–147.72)	2.29 (1.85–2.83)	1.61 (1.26–2.05)	1.68 (1.28–2.20)	1.63 (1.23–2.16)
Underweight			*P *<* *0.001	*P *<* *0.001	*P *<* *0.001	*P *<* *0.001
No	216	41.18 (36.04–47.06)	1	1	1	1
Yes	317	78.55 (70.36–87.69)	1.88 (1.58–2.24)	2.12 (1.76–2.54)	1.64 (1.36–1.98)	1.69 (1.35–2.12)
Missing	24	109.37 (73.31–163.18)	2.48 (1.68–3.65)	2.06 (1.31–3.25)	1.76 (1.11–2.78)	–
Period of follow-up			*P *<* *0.001	*P *<* *0.001	*P *<* *0.001	*P *<* *0.001
Pre-ART	200	70.14 (61.06–80.56)	1	1	1	1
ART <6 months	238	664.03 (584.81–753.98)	8.14 (6.70–9.90)	5.55 (4.50–6.84)	4.83 (3.88–6.01)	4.18 (3.21–5.44)
ART ≥6 months	119	18.92 (15.81–22.64)	0.28 (0.22–0.35)	0.19 (0.15–0.24)	0.31 (0.24–0.38)	0.31 (0.23–0.40)

HR, hazard rate ratio; PY, person-years of follow-up; SHR, sub-hazard rate ratio.

Two-year attrition was also higher amongst children of <2 years [244.00 compared with 164.65 per 1000 person-years in the 2- to 4-year group; and 151.40 per 1000 person-years in the 5- to 14-year group (Table3)]. Adjusted IR (aIR) = 2.10, 95% CI 1.86–2.37 and aIR = 1.28, 95% CI 1.13–1.44, respectively, compared with the 5- to 14-year group.

**Table 3:** Changes in associations between mortality and attrition over time stratified by age group

	<2 years		2–4 years		5–15 years	
	No. of deaths	Adjusted HR	No. of deaths	Adjusted HR	No. of deaths	Adjusted HR
Mortality						
2001–2004	28	0.73 (0.47–1.14)	35	2.21 (1.26–3.86)	74	2.46 (1.60–3.77)
2005–2007	93	0.95 (0.70–1.27)	67	2.03 (1.25–3.29)	75	1.92 (1.27–2.09)
2008–2010	116	1	25	1	44	1
Attrition						
2001–2004	88	0.80 (0.62–1.04)	130	1.17 (0.90–1.53)	207	1.14 (0.92–1.41)
2005–2007	231	0.85 (0.70–1.03)	208	0.99 (0.79–1.24)	234	1.63 (1.30–2.06)
2008–2010	266	1	143	1	192	1

*P*-value for likelihood ratio test for interaction between age group and year of programme entry was <0.001 for mortality and for attrition.

**Fig 2:**
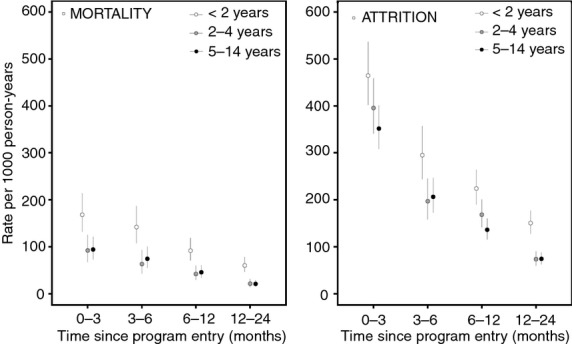
Mortality and attrition rates by age group and time since programme entry.

Restriction of analyses to patients with complete case data did not change the results for mortality or programme attrition. Rates of mortality and attrition were higher during the first 6 months of ART and gradually decreased over time after this period (Figure2).

### Other factors associated with programme attrition and mortality

Advanced clinical disease presentation (aHR = 1.68, 95% CI 1.28–2.20 for stage 3; and aHR = 2.47, 95% CI 1.80–3.38 for stage 4; compared with patients with stage 1), underweight (aHR = 2.12, 95% CI 1.76–2.54) and tuberculosis diagnosis (aHR = 1.61, 95% CI 1.26–2.05) were strongly associated with increased risk of mortality. Higher mortality was also observed during the first 6 months of ART and lower in the 6–24 months after ART initiation (aHR = 5.55, 95% CI 4.50–6.84; and aHR = 0.19, 95% CI 0.15–0.24, respectively, compared with the pre-ART period) and during early years of programme activity for children aged 2 years or more [aHR = 2.21, 95% CI 1.26–3.86 and aHR = 2.03, 95% CI 1.25–3.29 for the periods 2001–2004 and 2005–2007, respectively, compared with 2008–2010 in the 2–4 age group; and 2.46, 95% CI 1.60–3.77 and 1.92, 95% CI 1.27–2.09, respectively, in the 5–12 age group (Table3)]. The same associations were identified when competing risks regression, and complete case data analyses were performed. Nevertheless, the size of the effects estimated in sensitivity analyses was smaller for advanced clinical disease (clinical stages 3 and 4 and underweight) and for the period of follow-up on ART. And stronger effects were also observed for year of programme entry in the complete case analysis.

Similarly, higher risk of programme attrition was observed in patients with severe clinical disease (aHR = 1.75, 95% CI 1.51–2.03 for stage 3; and aHR = 3.06, 95% CI 2.56–3.66 for stage 4), underweight (aHR = 1.61, 95% CI 1.45–1.78) and tuberculosis diagnosis at programme entry (aHR = 1.31, 95% CI 1.12–1.54). The risk of attrition was also higher during the first 6 months of ART use and lower in the following 18 months (aHR = 2.43, 95% CI 2.16–2.74; and aHR = 0.09, 95% CI 0.08–0.11, respectively, compared with the follow-up period before ART start). Results from analyses restricted to patients with complete case data were similar (Table4), although the association with year of programme entry was only seen for the older age group of children (Table3).

**Table 4:** Association between attrition and selected individual-level factors

Factors	No. of deaths	Attrition rate per 1000 PY	All patient data		Complete case
			Crude IR	Adjusted IR	Adjusted IR
Site			*P *<* *0.001	*P *=* *0.003	*P *=* *0.005
Uganda	351	191.85 (172.80–213.01)	1	1	1
Malawi	870	163.14 (152.65–174.35)	0.85 (0.75–0.96)	0.91 (0.80–1.04)	0.94 (0.81–1.09)
Kenya – rural	287	215.54 (191.99–241.98)	1.12 (0.96–1.31)	1.03 (0.88–1.21)	1.10 (0.91–1.31)
Kenya – urban	191	189.87 (164.76–218.80)	0.99 (0.83–1.18)	1.26 (1.05–1.51)	1.30 (1.07–1.58)
Sex			*P *=* *0.22	*P *=* *0.13	*P *=* *0.57
Male	849	184.31 (172.32–197.14)	1	1	1
Female	850	173.70 (162.40–185.77)	0.94 (0.86–1.04)	0.93 (0.84–1.02)	0.97 (0.87–1.08)
Age group			*P *<* *0.001	*P *<* *0.001	*P *<* *0.001
5–14 years	633	151.40 (140.05–163.67)	1	1	1
2–4 years	481	164.65 (150.57–180.04)	1.09 (0.97–1.22)	1.28 (1.13–1.44)	1.29 (1.12–1.47)
<2 years	585	244.00 (225.01–264.59)	1.61 (1.44–1.80)	2.10 (1.86–2.37)	2.10 (1.82–2.41)
Clinical stage			*P *<* *0.001	*P *<* *0.001	*P *<* *0.001
1	352	141.80 (127.74–157.42)	1	1	1
2	219	151.37 (132.60–172.81)	1.07 (0.90–1.26)	1.26 (1.06–1.49)	1.24 (1.04–1.47)
3	476	203.72 (186.22–222.87)	1.44 (1.25–1.65)	1.75 (1.51–2.03)	1.70 (1.46–1.99)
4	246	327.02 (288.61–370.55)	2.31 (1.96–2.71)	3.06 (2.56–3.66)	3.07 (2.55–3.70)
Missing	406	163.57 (148.41–180.28)	1.15 (1.00–1.33)	1.11 (0.96–1.29)	–
Tuberculosis diagnosis			*P *<* *0.001	*P *<* *0.001	*P *=* *0.007
No	1474	170.47 (161.99–179.40)	1	1	1
Yes	225	263.71 (231.41–300.52)	1.55 (1.34–1.78)	1.31 (1.12–1.54)	1.26 (1.07–1.49)
Underweight			*P *<* *0.001	*P *<* *0.001	*P *<* *0.001
No	786	149.86 (139.75–160.72)	1	1	1
Yes	852	211.11 (197.40–225.78)	1.41 (1.28–1.55)	1.61 (1.45–1.78)	1.63 (1.45–1.83)
Missing	61	277.99 (216.30–357.29)	1.92 (1.50–2.46)	2.43 (1.83–3.22)	–
Period of follow-up			*P *<* *0.001	*P *<* *0.001	*P *<* *0.001
Pre-ART	974	341.56 (320.77–363.69)	1	1	1
ART <6 months	443	1235.98 (1126.09–1356.61)	3.62 (3.23–4.05)	2.43 (2.16–2.74)	2.39 (2.07–2.75)
ART ≥6 months	282	44.83 (39.90–50.39)	0.13 (0.11–0.15)	0.09 (0.08–0.11)	0.09 (0.08–0.11)

IR, incidence rate ratio; PY, person-years of follow-up.

## Discussion

In this study conducted amongst 6261 paediatric patients who were receiving HIV care in four large HIV programmes in sub-Saharan Africa over a 10-year period, fewer than 5% had a history of PMTCT prophylaxis use and medical referral was the mode of entry in two of five patients. Mortality and attrition during the first two years of follow-up in HIV care were two times higher amongst the youngest children than amongst those aged five to fourteen years. Advanced clinical disease presentation, underweight and tuberculosis diagnosis were strongly associated with poor patient outcomes. Higher rates were observed in earlier years of programme activity and during the first six months of ART use and lower in the following 18 months of therapy.

In our study, patients aged <2 years represented 27% of the total number of children accessing HIV care. This figure is lower than proportions reported in other studies, where patients aged <2 years represented almost 40% of the total number of children accessing HIV care (Sutcliffe *et al.* 2011; Munyagwa *et al.* 2012). And this highlights the limited access that young children have to HIV testing and counselling in the study area. Poor access is likely to be related to a combination of inadequate access to PMTCT services, limited diagnostic capacity, especially during early years of programme activity and/or poor linkage to HIV services after diagnosis.

Besides the technological barriers to earlier enrolment of children into HIV care, the poor integration of HIV and maternal child health services means that opportunities to detect infected mothers and their infants are often missed. Almost half of the total number of children included in our analysis entered HIV care via medical referral. This finding suggests that new strategies to expand access to early HIV testing are needed to bring children into care before they are sick. Approaches that engage maternal and child health services, integration of VCT in outpatient services to facilitate HIV testing of sick children, or community-based testing need to be further developed and evaluated. As new more effective PMTCT interventions such as the option B+ (provision of uninterrupted ART to pregnant women irrespective of patient eligibility for therapy) are implemented, it is expected that a minority of children born to mothers attending PMTCT services will acquire HIV infection, and therefore, most infants diagnosed with HIV will no longer be identified in PMTCT programmes.

The low proportion of children with documented exposure to prophylaxis for PMTCT of HIV in the programmes evaluated may reflect underreporting of prophylactic use, low rates of HIV infection amongst PMTCT users and/or sub-optimal access to PMTCT care. It also reflects the important challenges faced by care providers to implement effective PMTCT programmes in Africa. Given that, in the absence of ART use, approximately half of perinatally infected children die before the age of 2 years(Newell *et al.* 2004; Penazzato *et al.* 2012), it is likely that in areas covered by the programmes evaluated, many HIV-infected children died without ever being recognised as infected with HIV.

Mortality and attrition observed in these cohorts of children was higher than amongst older patients, a finding consistent with previous studies (Raguenaud *et al.* 2009; Sutcliffe *et al.* 2011; McNairy *et al.* 2012). It is now well accepted that there are significant benefits in terms of mortality and morbidity reduction and improved general psychomotor development associated with early rather than deferred ART (Violari *et al.* 2008; Goetghebuer *et al.* 2012; Laughton *et al.* 2012). However, as the results of this study show, in reality, there continues to be many missed opportunities or gaps to access, diagnose and treat young children with HIV early enough to achieve this reduction in risk. Globally, in 2010, data from 65 low- and middle-income countries showed that only an estimated 28% of HIV exposed infants had access to early infant diagnosis (WHO 2011). Improving access to point of care testing at 6 weeks of age as recommended by WHO would be a significant step forward and could lead to earlier treatment initiation and reduced mortality of infants infected *in utero* (Sherman 2012).

As for adults, studies have shown that a large proportion of children lost to follow-up (12–87% in African settings) may be unrecorded deaths (McGuire *et al.* 2012). Besides reflecting mortality, the particularly high lost to follow-up rate amongst infants is likely to reflect the difficulties faced by HIV-infected women with young children who need to deal with their own status and that of their child. Financial family constraints and parental disease or death related to HIV infection are also likely to lead to delays in paediatric care and contribute to increased mortality and dropouts. Adapted counselling strategies and support structures for women and their families are often not sufficiently in place (Hassan *et al.* 2012).

Children aged 5–14 years constitute an important group amongst those enrolled in HIV care. These ‘long-term survivors’ have particular needs that are usually not adequately met in resource-limited settings. Whilst they do not have the same mortality risk as younger children as shown by this study and others (Charlebois *et al.* 2010; Fenner *et al.* 2010), they face significant morbidities, including risk of poor physical and neurodevelopmental growth, poor sexual maturation and risk of chronic lung disease (Smith *et al.* 2012). Furthermore, from a social perspective, this group of children has a higher likelihood of being orphaned and often struggles with challenges in their home life as well as at school, which has implications for their adherence to treatment (Williams *et al.* 2006).

The observed decrease in mortality over time amongst children may be attributed to progressive enrolment of children at younger ages and at earlier stages of disease. In addition, it may also reflect improvement in clinical management as clinicians acquire more experience and as new guidelines including updated recommendations are published and implemented. In the early 2000s, there was little guidance on how to best care for children infected with HIV infection. The first WHO guideline specifically concerning the care of HIV positive children was published in 2006. After the publication of results from the CHER study, in 2008, WHO recommended treating all infants before the age of 1 year, regardless of clinical or immunological status (Violari *et al.* 2008; Puthanakit & Bunupuradah 2010; WHO 2010). The findings of our study show that, despite the achievements of recent years, there is still a need to reduce deaths and lost to follow-up amongst children through earlier HIV diagnosis, proper screening and treatment for opportunistic infections such as tuberculosis, earlier start of ART and adequate education and counselling for children and their caregivers to maximise retention in care. Progressively increasing the age threshold for the systematic initiation of therapy of children older than one year might be a pragmatic strategy to simplify paediatric HIV care provision and improve patient outcomes by reducing delays in ART start, mortality and lost to follow-up rates.

Finally, advanced clinical disease, underweight and tuberculosis diagnosis at enrolment were strongly associated with increased risk of mortality or attrition. These findings are similar to those of previous studies (Munyagwa *et al.* 2012; Sutcliffe *et al.* 2011; Raguenaud *et al.* 2009; Fenner *et al.* 2010; Cross Continents Collaboration for Kids (3Cs4kids) Analysis & Writing Committee 2008; Wamalwa *et al.* 2012). Tuberculosis is an opportunistic infection frequently associated with HIV infection in adults and children and associated with increased mortality (Kyeyune *et al.* 2010; Bakeera-Kitaka *et al.* 2011). However, the burden of tuberculosis in both HIV-infected and HIV-uninfected children is significantly underestimated due to the difficulties of diagnosis (Kyeyune *et al.* 2010; Bakeera-Kitaka *et al.* 2011). Paediatric HIV infection is associated with a 20-fold increased risk of developing active tuberculosis (Hesseling *et al.* 2009). In our analysis, 9.8% of all children were diagnosed with tuberculosis at the time of enrolment in HIV care. Based on results from other studies conducted amongst adults and children living in the same geographical areas, this figure is likely to underestimate the true burden of disease amongst children (Shah *et al.* 2009; Bakeera-Kitaka *et al.* 2011). Published research (Bakeera-Kitaka *et al.* 2011; Marais *et al.* 2011) suggests that undiagnosed tuberculosis is likely to have contributed to the high mortality observed before and in the 6 months following ART start.

Several limitations need to be considered when interpreting the results of this evaluation. First, we used electronic health records with missing CD4 counts for most of the children and probable underreporting of PMTCT exposure. Nevertheless, the results obtained in the complete case data analysis, although generally of smaller size, were consistent with those estimated in primary analyses. Second, as shown in previous studies (McGuire *et al.* 2010; Desmonde *et al.* 2011), it is likely that many of the children lost to follow-up were unreported deaths, resulting in underestimation of mortality. However, competing risk analyses and those examining the association between baseline factors and retention in care were consistent with results of primary analyses. Third, we cannot exclude residual confounding by unmeasured factors such as information on family support and caregiver, which are likely to be associated with mortality (Sutcliffe *et al.* 2011). Finally, we excluded 6% of patients who had a unique clinic visit and were more likely to have advanced clinical disease, be underweight and to have died (Table S1). This might have resulted in some underestimation of study effects.

## Conclusion

The results of this large study conducted under programmatic conditions and over a 10-year period add to our understanding of the challenges faced in the initial years of treating paediatric HIV infection in sub-Saharan African and of the evolution of public health programmes over time. Whilst good progress has been made in generating evidence through field research, creating practical guidance and developing adapted diagnostic tools and drug formulations for children, there remains significant work still to do. This study highlights the need to increase access, diagnose and provide early HIV care to children and to accelerate ART initiation for those eligible. It also stresses the importance of finding better ways to increase HIV testing of high-risk group children (e.g. amongst chronically ill and/or malnourished), effectively screen for and treat opportunistic infections, especially tuberculosis, and of ensuring that education and support are adapted to the needs of the children and their family.

## Supporting Information

Table S1 Characteristics of patients included and excluded from the analyses measured at program entry.
