# Supplementary material for: Paediatric HIV care in sub-Saharan Africa: clinical presentation and 2-year outcomes stratified by age group
Source: Trop Med Int Health. 2013 Jun 20;18(9):1065–74. doi: 10.1111/tmi.12142 (PMC4285230; doi:10.1111/tmi.12142)
Supplement: Table S1 — Characteristics of patients included and excluded from the analyses measured at program entry. [file tmi0018-1065-SD1.doc]

**SUPLEMENTAL DIGITAL CONTENT**

**Table S1. Characteristics of patients included and excluded from the analyses measured at program entry**

| **Characteristics** | **Included (>1 visit)**  **N=6261** | **Excluded (unique visit)**  **N=421** | **Total**  **N=6682** |
| --- | --- | --- | --- |
| **Female, n (%)** | 3197 (51.1) | 212 (50.4) | 3409 (51.0) |
| **Age group, n (%)** |  |  |  |
| <2 years | 1698 (27.1) | 133 (31.6) | 1831 (27.4) |
| 2-4 years | 1885 (30.1) | 129 (30.6) | 2014 (30.1) |
| 5-14 years | 2678 (42.8) | 159 (37.8) | 2837 (42.5) |
| **Mode of entry, n (%)** |  |  |  |
| Medical referral | 2309 (42.7) | 174 (47.3) | 2483 (43.0) |
| Volunteer counseling and testing | 2541 (47.0) | 153 (41.6) | 2694 (46.6) |
| Other | 558 (10.3) | 41 (11.1) | 599 (10.4) |
| Missing | 853 | 53 | 906 |
| **History of PMTCT prophylaxis use, n (%)** | 106 (1.7) | 3 (0.7) | 109 (1.6) |
| **Clinical stage, n (%)** |  |  |  |
| 1 | 1588 (33.9) | 76 (23.4) | 1664 (33.2) |
| 2 | 928 (19.8) | 36 (11.1) | 964 (19.3) |
| 3 | 1576 (33.7) | 125 (38.5) | 1701 (34.0) |
| 4 | 590 (12.6) | 88 (27.1) | 678 (13.5) |
| Missing | 1579 | 96 | 1675 |
| **Tuberculosis diagnosis, n (%)** | 613 (9.8) | 33 (7.8) | 646 (9.7) |
| **Median weight, kg [IQR]** | 12.0 [8.5-19.0] | 10.0 [7.1-17.0] | 12.0 [8.3-18.3] |
| Missing, n (%) | 11 (0.2) | 31 (7.4) | 42 (0.6) |
| **Underweight, n (%)** |  |  |  |
| No | 3329 (54.7) | 139 (37.5) | 3468 (53.7) |
| Yes | 2761 (45.3) | 232 (62.5) | 2993 (46.3) |
| Missing | 171 | 50 | 221 |
| **Dead, n (%)** | 557 (8.9) | 61 (14.5) | 618 (9.2) |
| **Lost to follow-up, n (%)** | 1142 (18.2) | 353 (83.8) | 1495 (22.4) |

Note: IQR, interquartile range; PMTCT, prevention of mother to child transmission of HIV infection
